# Healthcare-associated infections by multidrug-resistant bacteria in Andalusia, Spain, 2014 to 2021

**DOI:** 10.2807/1560-7917.ES.2023.28.39.2200805

**Published:** 2023-09-28

**Authors:** Nicolás Francisco Fernández-Martínez, Mario Rivera-Izquierdo, Rocío Ortiz-González-Serna, Virginia Martínez-Ruiz, Pablo Lardelli-Claret, Adrián Hugo Aginagalde-Llorente, María del Carmen Valero-Ubierna, María Auxiliadora Vergara-Díaz, Nicola Lorusso

**Affiliations:** 1 Unidad de Gestión Clínica Interniveles de Prevención, Promoción y Vigilancia de La Salud, Hospital Universitario Reina Sofía, Cordova, Spain; 2 Maimonides Institute for Research in Biomedicine of Cordoba (IMIBIC), Cordova, Spain; 3 Department of Preventive Medicine and Public Health, University of Granada, Avda. de la Investigación n°11, Granada, Spain; 4 Unidad de Gestión Clínica Interniveles de Prevención, Promoción y Vigilancia de La Salud, Hospital Universitario San Cecilio, Granada, Spain; 5 Subdirección de Promoción de la Salud y Prevención, Dirección General de Salud Pública, Ministerio de Sanidad, Madrid, Spain; 6 Dirección General de Salud Pública y Ordenación Farmacéutica, Consejería de Salud y Consumo, Junta de Andalucía, Seville, Spain

**Keywords:** epidemiological surveillance, healthcare-associated infections, multidrug resistance, infectious diseases, spatial epidemiology

## Abstract

**Background:**

Multidrug-resistant (MDR) bacteria are among chief causes of healthcare-associated infections (HAIs). In Spain, studies addressing multidrug resistance based on epidemiological surveillance systems are lacking.

**Aim:**

In this observational study, cases of HAIs by MDR bacteria notified to the epidemiological surveillance system of Andalusia, Spain, between 2014−2021, were investigated. Notified cases and their spatiotemporal distribution were described, with a focus on social determinants of health (SDoH).

**Methods:**

New cases during the study period of HAIs caused by extended-spectrum β-lactamase (ESBL)-/carbapenemase-producing Enterobacterales, MDR *Acinectobacter baumannii*, MDR *Pseudomonas aeruginosa* or meticillin resistant *Staphylococcus aureus* were considered. Among others, notification variables included sex and age, while socio-economic variables comprised several SDoH. Cases’ spatial distribution across municipalities was assessed. The smooth standardised incidence ratio (sSIR) was obtained using a Bayesian spatial model. Association between municipalities’ sSIR level and SDoH was evaluated by bivariate analysis.

**Results:**

In total, 6,389 cases with a median age of 68 years were notified; 61.4% were men (n = 3,921). The most frequent MDR bacteria were ESBL-producing Enterobacterales (2,812/6,389; 44.0%); the main agent was *Klebsiella* spp. (2,956/6,389; 46.3%). Between 2014 and 2021 case numbers appeared to increase. Overall, up to 15-fold differences in sSIR between municipalities were observed. In bivariate analysis, there appeared to be an association between municipalities’ sSIR level and deprivation (p = 0.003).

**Conclusion:**

This study indicates that social factors should be considered when investigating HAIs by MDR bacteria. The case incidence heterogeneity between Andalusian municipalities might be explained by SDoH, but also possibly by under-notification. Automatising reporting may address the latter.

**Key public health message**

**What did you want to address in this study?** 

This study aimed to use data from the epidemiological surveillance system of Andalusia, Spain, to investigate healthcare-associated infections caused by multidrug-resistant bacteria. We wanted to explore the temporal and spatial trends of occurrences of such infections in the region between 2014 and 2021. We also wondered if social determinants of health affected the epidemiology of infections.

**What have we learnt from this study?** 

Healthcare-associated infections due to multidrug-resistant bacteria seemed to increase during the study period. The most frequent bacteria causing infections were so-called ESBL-producing Enterobacterales. A geographical variation in incidence of infections was observed and incidence appeared to relate to the index of deprivation of Andalusian municipalities. There were indications of possible underreporting of infections in the region.

**What are the implications of your findings for public health?** 

The apparent increase in healthcare-associated infections by multidrug-resistant bacteria during the study period is concerning, as this may imply that therapeutic options for these infections could become more limited in the future. When studying the transmission of multidrug-resistant bacteria, considering socioeconomic factors is important. To ameliorate the epidemiological surveillance performance, notifications need to improve in the region; for this, their automatisation might help.

## Introduction

Antimicrobial resistance is the ability of microorganisms to withstand the effect of antimicrobial agents. Antibiotic resistance, its principal constituent, refers to the ability of bacteria to resist the action of antibiotics through a variety of determinants, which bacteria attempt to accumulate in a phenomenon coined as ‘genetic capitalism’ [1]. This may result in multidrug resistance, that is, non-susceptibility to at least one agent in three or more antimicrobial categories. While also present in the community, multidrug-resistant (MDR) bacteria are among the chief causes of infections in the healthcare setting [2], including in hospitals, healthcare centres and nursing homes, which are covered under the term healthcare-associated infections (HAIs).

Multidrug resistance incidence has been rising for several decades worldwide. In Europe, although rates of MDR isolates have stabilised, incidences of vancomycin resistance in *Enterococcus faecium*, and carbapenem resistance in *Escherichia coli* and *Klebsiella pneumoniae* have significantly increased throughout the period 2016–2020 [3]. Compared with their non-resistant counterparts, higher mortality rates have been found for infections by antibiotic-resistant bacteria [4]. Despite considerable variation according to geographical location – in Europe, between 50 and 80 annual deaths per 100,000 population –, the global burden of antibiotic resistance in 2019 has been estimated at 5 million associated deaths annually, thereby placing it as a leading cause of death [5]. Considering the problem, the World Health Organization published, already in 2017, a list of pathogens with certain resistances, to prioritise for research and development of new antibiotics. The critical priority group comprised Enterobacterales, *Acinetobacter baumannii* and *Pseudomonas aeruginosa* with carbapenem resistance, and Enterobacterales with third-generation cephalosporin resistance [6].

Although bacteria can be intrinsically resistant to antibiotics, antibiotic resistance has been undoubtedly catalysed by human beings. The introduction of antibiotics improved drastically the prognosis of most bacterial infections but was not without risks. Ample evidence supports that overuse and misuse of antibiotics are major drivers of antibiotic resistance [7], as shown in experimental [8], observational [9] and ecological studies [10], hence the development of antimicrobial stewardship programmes. These programmes, which aim to optimise antibiotic prescribing, have demonstrated their effectiveness at reducing the incidence of infection and colonisation with antibiotic-resistant bacteria in hospital inpatients, especially when coupled with infection control measures.

However, there is more to antibiotic resistance than antibiotic consumption. The risk of development of resistance is also shaped by age and/or underlying diseases of those receiving the antibiotic, as well as psychological, social and environmental factors. Social determinants of health (SDoH), broadly defined as ‘the full set of social conditions in which people live and work’, can be divided into structural (e.g. education) and intermediary determinants (e.g. material circumstances). Poverty [11], lower socioeconomic status [12], deprivation at the neighbourhood level [13], and low public healthcare expenditure [14] have been associated with a higher risk of antimicrobial resistance occurrence. Though consistent with previous research on infectious diseases, the evidence for urbanicity is limited. Some scientists have posited the need for an approach to address antibiotic resistance including sociocultural determinants. Further, the World Health Organization recently acknowledged the social dimensions of antimicrobial resistance [15].

Surveillance systems attempt to monitor communicable diseases that threaten human health, including those involving antimicrobial resistance. The European Antimicrobial Resistance Surveillance Network (EARS-Net) integrates data from countries in the European Union/European Economic Area. In 2016, the Spanish Epidemiological Surveillance Network (RENAVE) developed a specific protocol concerning notification of HAIs by MDR bacteria and other organisms of epidemiological interest. In southern Spain, an extensive surveillance system integrated within RENAVE has been monitoring notifiable diseases since 1996: the Epidemiological Surveillance System of Andalusia (SVEA). Nonetheless, few studies have described or analysed MDR data from epidemiological surveillance systems in Spain and fewer have done so considering social factors.

The objective of this study was to comprehensively describe the distribution of cases of HAIs by MDR bacteria notified to the SVEA over an 8-year period, focusing on the SDoH.

## Methods

### Setting and study design

Andalusia is the most populated region of Spain with more than 8.5 million inhabitants across eight provinces that include 785 municipalities. The Andalusian health system comprises 33 health districts and, as part of the national healthcare system, universal healthcare coverage is provided to any of its residents. However, annual public per capita expenditure on healthcare is the lowest in the country (€1,398 in 2020). 

We conducted a descriptive study of cases of HAIs by MDR bacteria during the period 2014–2021 in Andalusia. The study investigated the characteristics of cases and their distribution in the region (ecological design), with municipalities as the unit of analysis.

### Data sources and variables

Data at the individual level were obtained from the SVEA registries [16], which store case-based epidemiological information; of note, non-administrative data are documented exclusively by hand. SVEA registries combine hospital data from preventive medicine units and community data from primary care epidemiology units; long-term care facility data are collected as a separate category and are considered to originate from the community. However, as epidemiological surveillance of MDR bacteria in the community (primary care) is currently under implementation (and planned to be fully integrated in the medium term), data in this study essentially came from preventive medicine units. The variables included were sex (male/female), age, causative agent, mechanism(s) of resistance, infection site, source of infection, department of diagnosis (when the source was a hospital), outbreak association, georeferenced data (X and Y coordinates) for the place of residence, province, and year.

Regional data were taken from the National Statistics Institute (INE), the Institute of Statistics and Cartography of Andalusia (IECA), the deprivation index by census sections developed by the Spanish Society of Epidemiology [17] and the quarterly reports from the antimicrobial stewardship programme in Andalusia – Institutional Programme for the Prevention and Control of Healthcare-Associated Infections and Appropriate Use of Antimicrobials (PIRASOA) [18]. At the municipality level, demographic variables were the total population as of 2018 (available for 99.1% of municipalities), and the degree of urbanisation according to the ‘DEGURBA’ classification as of 2016, which categorises areas as densely populated’ (cities), with intermediate population density or thinly populated (rural) [19]. The main socioeconomic variable was the national deprivation index (2011) developed by the Spanish Society of Epidemiology, which consists of six indicators related to education, occupation, and housing conditions (available for 98.2% (771/785) of municipalities). With a mean of zero, its values range from − 2.6 (least deprived) to + 4.9 (most deprived). We also included the annual average gross income per capita as of 2015 (available for 98.9% (776/785) of municipalities) and the Gini coefficient [20,21] as of 2015 (available for 98.5% (773/785) of municipalities [22]). Last, outpatient antibiotic consumption (2014–2021), representing ca 90% of the total antibiotic consumption, was included by health district. Of note, PIRASOA reports group together data from certain districts, so antibiotic consumption was reported according to 28 subsets of the Andalusian health system instead of 33.

### Definitions

#### Healthcare associated infections

HAIs were defined as any clinical syndrome caused by an infectious agent or its toxins, when (i) evidence of infection first appears at least 48 hours after hospital admission (hospital-onset); or when (ii) one or more risk factors associated with healthcare are present: history of stay in a healthcare facility or long-term care facility, specialised home healthcare, dialysis or day hospital treatment, previous surgery or invasive procedures, such as central venous catheterisation (community-onset). There were two exceptions to this rule. On the one hand, carbapenemase-producing Enterobacterales (CPE), for which cases of colonisation (i.e. evidence of microorganisms without infectious syndrome) meeting any of these two criteria were also classified as HAIs. On the other, in the event of surgical site infections, the period considered was 30 days since the date of surgery, and 90 days for certain operative procedures (e.g. implant surgeries) [23].

#### Multidrug-resistant bacteria

In accordance to the SVEA-adapted RENAVE protocol for HAIs by MDR bacteria [24], these were defined as: (i) CPE, (ii) extended-spectrum β-lactamase (ESBL) producing Enterobacterales, (iii) meticillin-resistant *Staphylococcus aureus* (MRSA), (iv) MDR *Acinetobacter baumannii* and (v) MDR *P. aeruginosa*. It should be noted that notification of HAIs by MDR *P*. *aeruginosa*, as well as colonisation by CPE, were not included in the protocol until 2018.

#### Outpatient antibiotic consumption

Outpatient antibiotic consumption was defined as the added non-hospital consumption of the following antibiotics: amoxicillin, amoxicillin−clavulanic acid, cefadroxil, ceftibuten, cefuroxime, cefixime, ciprofloxacin, levofloxacin, moxifloxacin, trimethoprim/sulfamethoxazole, clindamycin, erythromycin, clarithromycin, azithromycin, and fosfomycin [18]. Outpatient antibiotic consumption was quantified in defined daily doses (DDD) per 1,000 inhabitants per day at the health district level.

### Statistical analyses

Quantitative variables, tested for normal distribution by the Shapiro−Wilk test, were described as mean and standard deviation if normally distributed, or as median and interquartile range (IQR) if not. Qualitative variables were described as absolute counts and percentages. In the bivariate analysis, categorical variables were compared using Pearson’s Chi-squared test or Fisher’s exact test, when appropriate, whereas quantitative variables were compared using Kruskal−Wallis H test.

At the individual level, we graphically described the temporal distribution of HAIs due to MDR bacteria stratified by causative agents and mechanisms of resistance. Assigning cases to their municipality of residence, which required georeferenced data to be available, allowed us to investigate their geographical distribution.

At the municipality level, we assessed the standardised incidence ratio (SIR): the quotient between observed and expected new cases, setting as reference the average ratio across municipalities. In this study, the SIR was obtained through indirect standardisation by sex and age. To assess the overall spatial autocorrelation, the global Moran’s I was calculated, and its p value was obtained through Monte Carlo simulations. Next, we employed the Bayesian spatial model proposed by Besag, York and Mollié [25] to smooth the SIR in order to avoid the effect of small areas. This model uses Poisson regression with random effects, which represents the heterogeneity of each geographic unit and the spatial contiguity, based on an autoregressive conditional model (CAR):

*Yi ~ Poisson(E_i_ λ_i_)*

*log(λ_i_)* = *α* + *h_i_* + *b_i_* [26].

In the equation shown, *λ_i_* is the relative risk (RR) in area *i*; *Y_i_* is the number of new cases in area *i*; *α* is the constant; *E_i_* is the expected number of new cases; *h_i_* is a spatial structured component modelled with a CAR distribution; and *b_i_* is an unstructured spatial effect (i.e. an ordinary random-effects component for non-spatial heterogeneity among municipalities). Although *λ_i_* is usually interpreted as the RR in area *i*, no independent variable was introduced in this model, so we reckon that ‘smoothed SIR’ (sSIR) is a more appropriate term in this case. The sSIR *λ_i_*, along with its 95% credible intervals, quantifies whether area *i* has equal (= 1), higher (> 1) or lower (< 1) incidence than the average incidence in the standard population.

Then, we used choropleth maps to represent the sSIR of HAIs by MDR bacteria, overall and according to the main mechanisms of resistance. To enhance visualisation, we created an interactive map and an interactive table. These are available in English and in Spanish, so that they can be more accessible for public health professionals in Spain.

At the health district level, we graphically described the temporal distribution of outpatient antibiotic consumption including a generalised additive model to smooth seasonal variation, and we described its spatial distribution using a choropleth map.

The statistical analyses were performed with the R software version 4.2.0 [27]. We used the packages sf, SpatialEpi and R-INLA to build the spatial model, ggplot2 to make the plots and Shiny to create the interactive content.

## Results

### Cases’ characteristics and temporal variations in incidence of infections according to the pathogens’ resistance mechanisms

A total of 6,389 cases of HAIs by MDR bacteria were notified over the period 2014–2021. Their characteristics are shown in Table 1. Median age was 68.0 years (range: 0–102) and cases were predominantly men (61.4%). Among all cases, most cases with available data on the source of infection, had hospitals as the source (4,178/6,389; 65.4%), with internal medicine/infectious diseases (864/6,389; 13.5%) and adult intensive care units (844/6,389; 13.2%) as the main departments of diagnosis. While those departments were responsible for the MDR bacteria detection, it should be mentioned that the study design did not allow considering them as the actual source of infection. Also of note, is that data on the department of diagnosis were missing for half of cases. Concerning the causative agents, *Klebsiella* spp. (2,956/6,389; 46.3%) – essentially *K. pneumoniae* – were the most frequent, followed by *S. aureus* (18.5%) and *E. coli* (1,074/6,389; 16.8%), as presented in the Supplementary Material S1 and in Table 1. ESBL-production in Enterobacterales constituted the most frequent mechanism of resistance (44.0%), while carbapenemase production in Enterobacterales and meticillin-resistance in *S. aureus* were detected in 26.0% and 18.5% of cases, respectively.

**Table 1:** Characteristics of cases of healthcare-associated infections by multidrug-resistant bacteria, Andalusia, Spain, 2014–2021 (n = 6,389 cases)

Characteristic	Values	
**Sex**	**Number**	**Percentage**
Men	3,921	61.4
Women	2,468	38.6
**Age in years**	**Median**	**IQR**
Age	68.0	56.0–77.0
**Age groups in years**	**Number**	**Percentage**
0–9	147	2.3
10–19	63	1.0
20–29	108	1.7
30–39	227	3.6
40–49	499	7.8
50–59	958	15.0
60–69	1,479	23.1
70–79	1,656	25.9
80–89	1,051	16.5
≥ 90	201	3.1
**Mechanisms of resistance and causative agent**	**Number**	**Percentage^a^**
**ESBL-producing Enterobacterales**	**2,812**	**44.0**
*Klebsiella* spp.	1,763	27.6
*Escherichia coli*	1,001	15.7
*Enterobacter cloacae*	25	0.4
*Proteus mirabilis*	17	0.3
Other	3	0.0
Missing data	3	0.0
**Carbapenemase-producing Enterobacterales**	**1,658**	**26.0**
*Klebsiella* spp.	1,193	18.7
*Enterobacter cloacae*	334	5.2
*Escherichia coli*	73	1.1
*Citrobacter freundii*	26	0.4
*Proteus mirabilis*	10	0.2
*Serratia marcescens*	6	0.1
Other	7	0.1
Missing data	9	0.1
**Meticillin-resistant *Staphylococcus aureus***	**1,181**	**18.5**
**Multidrug-resistant *Acinetobacter baumannii***	**721**	**11.3**
**Multidrug-resistant *Pseudomonas aeruginosa***	**265**	**4.1**
**Carbapenemase-producing *P. aeruginosa***	**63**	**1.0**
**Other mechanisms of resistance**	**202**	**3.2**
**Infection site or type**	**Number**	**Percentage**
Urinary tract infection	821	12.9
Bacteraemia	468	7.3
Non-pneumonic respiratory tract infection	325	5.1
Surgical site infection	268	4.2
Skin and soft tissue infection	247	3.9
Pneumonia	168	2.6
Gastrointestinal infection	29	0.5
Osteoarticular infection	21	0.3
Infective endocarditis/vascular infection	9	0.1
Reproductive tract infection	8	0.1
Other site	113	1.8
None (colonisation)	593	9.3
Missing data	3,319	51.9
**Source of infection**	**Number**	**Percentage**
Hospital in which the infection was diagnosed	4,043	63.3
Hospital, other	135	2.1
Long-term care facility (e.g. nursing home)	131	2.1
Missing data	2,080	32.6
**Department of diagnosis**	**Number**	**Percentage**
Internal medicine/infectious diseases	864	13.5
Adult intensive care unit	844	13.2
General and digestive surgery	350	5.5
Traumatology and orthopaedic surgery	226	3.5
Neurosurgery	108	1.7
Nephrology	97	1.5
Urology	96	1.5
Haematology	83	1.3
Paediatric/neonatal intensive care unit	80	1.3
Cardiovascular/thoracic surgery	79	1.2
Gastroenterology	79	1.2
Other	272	4.3
Missing data	3,211	50.3
**Outbreak association**	**Number**	**Percentage**
Outbreak association	1,110	17.4
No outbreak association	5,279	82.6
**Province**	**Number**	**Percentage**
Malaga	1,414	22.1
Granada	1,406	22.0
Cadiz	922	14.4
Seville	907	14.2
Jaen	767	12.0
Cordova	356	5.6
Almeria	293	4.6
Huelva	232	3.6
Missing data	92	1.4
**Year**	**Number**	**Percentage**
2014	220	3.4
2015	559	8.7
2016	705	11.0
2017	735	11.5
2018	832	13.0
2019	914	14.3
2020	757	11.8
2021	1,667	26.1

ESBL: extended-spectrum β-lactamase; IQR: interquartile range; spp.: species.

^a^ It should be noted that the proportions of each mechanism of resistance add up to more than 100%, as two or more mechanisms coexisted in certain cases (e.g. infections caused by ESBL-producing Enterobacterales and carbapenemase-producing *Escherichia coli*).

Data are presented as absolute frequency (number) and relative frequency (percentage) for qualitative variables, and as median and interquartile range for quantitative variables.

Of the 1,711 cases caused by carbapenemase-producing bacteria, specific carbapenemases were available in laboratory tests in 89.2% (n = 1,527) of them. As some cases were infected or colonised by more than one carbapenemase-producing species, the 1,527 cases corresponded to a total of 1,586 different carbapenemases/carbapenemase-producing species. The most frequent carbapenemases were OXA-48 (847/1,586; 53.4%), followed by KPC (450/1,586; 28.4%) and VIM (217/1,586; 13.7%); Table 2 shows the distribution of carbapenemases and their association with ESBL production. Among the total 6,389 cases, the contributions of multidrug resistance in *A. baumannii* and *P. aeruginosa* together accounted for 15.4% (Table 1). Regarding infection type, information was missing for 52% of cases. Among all cases, the most common HAI was urinary tract infection (821/6,389; 12.9%), followed by bacteraemia (468/6,389; 7.3%) and non-pneumonic respiratory tract infection (325/6,389; 5.1%). Nearly one in five cases (17.4%) was associated to an outbreak. 

**Table 2:** Carbapenemases identified by laboratory tests in cases of healthcare-associated infections by multidrug-resistant bacteria, Andalusia, Spain, 2014–2021 (n = 1,586 carbapenemases)

Carbapenemase	Number without ESBL	Number with ESBL	Number overall
**Type A**	430	20	450
**KPC alone**	**421**	**18**	**439**
**KPC and other carbapenemases**	**9**	**2**	**11**
KPC + OXA-48	5	1	6
KPC + VIM	4	1	5
**Type B**	268	20	288
**VIM alone**	**174**	**7**	**181**
**VIM and other carbapenemases**	**34**	**2**	**36**
VIM + OXA-48	29	1	30
VIM + KPC	3	1	4
VIM + NDM	1	0	1
VIM + IMP	1	0	1
**IMP alone**	**31**	**1**	**32**
**IMP and other carbapenemases**	**3**	**1**	**4**
IMP + OXA-48	1	1	2
IMP + VIM	1	0	1
IMP + NDM	1	0	1
**NDM alone**	**22**	**8**	**30**
**NDM and other carbapenemases**	**3**	**1**	**4**
NDM + OXA-48	1	1	2
NDM + IMP	1	0	1
NDM + VIM	1	0	1
**AIM alone**	**1**	**0**	**1**
**AIM and other carbapenemases**	**0**	**0**	**0**
**Type D**	670	178	848
**OXA-48 alone**	**632**	**173**	**805**
**OXA-48 and other carbapenemases**	**38**	**4**	**42**
OXA-48 + VIM	31	1	32
OXA-48 + KPC	5	1	6
OXA-48 + IMP	1	1	2
OXA-48 + NDM	1	1	2
**OXA-23 alone**	**0**	**1**	**1**
**OXA-23 and other carbapenemases**	**0**	**0**	**0**
**Total**	1,368	218	1,586

AIM: antibiotic inactivation mechanism; ESBL: extended-spectrum β-lactamase; IMP: imipenemase; KPC: *Klebsiella pneumoniae* carbapenemase; OXA: oxacillinase; NDM: New Delhi metallo-β-lactamase; VIM: Verona Integron-encoded Metallo-β-lactamase.

Data are presented as absolute frequency for qualitative variables.

There were 1,711 cases of healthcare-associated infections by carbapenemase-producing Enterobacterales or *Pseudomonas aeruginosa*. The carbapenemases presented correspond to 1,527 of those cases. In the remainder (184 cases), the specific carbapenemases involved were unknown (labelled in laboratory tests as ‘carbapenemase-producing’ without specification).

**Figure 1:**
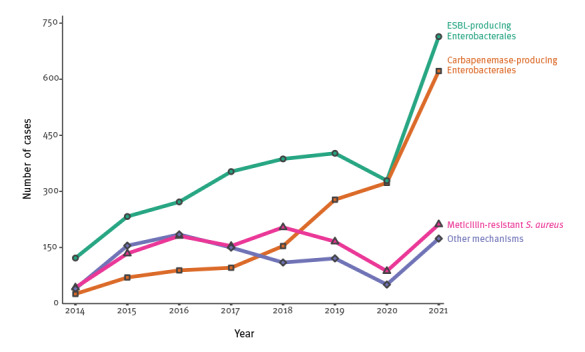
Temporal distribution of cases of healthcare-associated infections by multidrug-resistant bacteria, according to the mechanism of resistance, Andalusia, Spain, 2014–2021 (n = 6,637 mechanisms)^a^

ESBL: extended-spectrum β-lactamase; MDR: multidrug resistant; *S. aureus*: *Staphylococcus aureus.*

^a^ The 6,637 mechanisms correspond to 2,812 cases with identification of ESBL-production, 1,658 cases with carbapenemase-production, 1,181 with MRSA, and 986 with other mechanisms of resistance (e.g. MDR *P. aeruginosa*).

Over the study period, the frequency of HAIs by some MDR bacteria, mainly Enterobacterales, seemed to increase (except in 2020) and a particularly sharp rise was observed in 2021. The number of cases involving *K. pneumoniae* in particular appeared to show the largest increase over the study period, whereas those involving *E. cloacae* augmented in the last 2 years as illustrated in the Supplementary Material S1. In terms of the mechanism of resistance, the incidence of ESBL and especially carbapenemase production seemed to increase (Figure 1); however, with regard to Enterobacterales, 35.8% (n = 593) of the 1,658 cases with detection of CPE corresponded to colonisation. Moreover, the spatial distribution of cases of HAIs with MDR bacteria varied widely, ranging from less than 5% in some provinces (Huelva, Almeria) to more than 20% in others (Malaga, Granada) as shown in Table 1.

### Socio-demographic features of cases’ residence area and infection incidence

Georeferenced data were missing in 22.0% of cases (n = 1,407). As a result, at the municipality level, only 4,982 cases were considered (including 2,202 cases of HAIs by ESBL-producing Enterobacterales, 1,375 by CPE and 866 by MRSA). Table 3 shows the characteristics of the 785 municipalities studied. Although 10 cities gathered a third of the population in Andalusia, as described in Supplementary Material S2, most municipalities (n = 521; 66.4%) had a total population below 5,000 inhabitants and were classified as rural areas (67.8%). A vast majority pertained to the highest quintile of deprivation (73.5%); annual gross income per capita had a median of 8,559€ (IQR: 7,865–9,381€) and median Gini coefficient was 0.31 (IQR: 0.29–0.34). At the health district level, mean outpatient antibiotic consumption was 16.7 DDD per 100,000 inhabitants per day (range: 4.8–39.2) and steadily decreased until 2020, when a turning point was observed as plotted in the Supplementary Material S3.

**Table 3:** Socio-demographic characteristics of municipalities where cases resided, according to level of incidence of healthcare-associated infections by MDR bacteria, Andalusia, Spain, 2014–2021 (n = 785 municipalities)

Characteristic	All municipalities (n = 785)		Very low incidence sSIR ≤ 0.5 (n = 141)		Low incidence sSIR > 0.5 to ≤ 1.0 (n = 260)		High incidence sSIR > 1.0 to ≤ 2.0 (n = 297)		Very high incidence sSIR > 2.0 (n = 87)		p value
Number of inhabitants	Number	%	Number	%	Number	%	Number	%	Number	%	p value
1–999	216	27.5	46	32.6	54	20.8	89	30.0	27	31.0	0.338^a^
1,000–4,999	305	38.9	53	37.6	107	41.2	112	37.7	33	37.9	
5,000–19,999	175	22.3	27	19.1	57	21.9	68	22.9	23	26.4	
20,000–49,999	53	6.8	9	6.4	25	9.6	17	5.7	2	2.3	
≥ 50,000	29	3.7	5	3.5	14	5.4	8	2.7	2	2.3	
Unknown	7	0.8	1	0.7	3	1.2	3	1.0	0	0.0	
Degree of urbanisation	Number	%	Number	%	Number	%	Number	%	Number	%	p value
Rural areas	532	67.8	101	71.6	164	63.1	212	71.4	55	63.2	0.322^a^
Intermediate density areas	208	26.5	34	24.1	78	30.0	71	23.9	25	28.7	
Cities	45	5.7	6	4.3	18	6.9	14	4.7	7	8.0	
Deprivation index	Median	IQR	Median	IQR	Median	IQR	Median	IQR	Median	IQR	p value
Municipalities with available data	1.29	0.82–1.68	1.19	0.74–1.49	1.23	0.87–1.60	1.40	0.89–1.78	1.43	0.64–1.93	0.003^b^
Municipalities with no data	Number	%	Number	%	Number	%	Number	%	Number	%	p value
	14	1.8	1	0.7	5	1.9	5	1.7	3	3.4	NA
Deprivation index quintiles	Number	%	Number	%	Number	%	Number	%	Number	%	p value
Q1 (least deprived)	4	0.5	1	0.7	1	0.4	2	0.7	0	0.0	1.000^c^
Q2	20	2.5	2	1.4	7	2.7	8	2.7	3	3.4	
Q3	44	5.6	7	5.0	13	5.0	13	4.4	11	12.6	
Q4	126	16.1	34	24.1	38	14.6	43	14.5	11	12.6	
Q5 (most deprived)	577	73.5	96	68.1	196	75.4	226	76.1	59	67.8	
Unknown	14	1.8	1	0.7	5	1.9	5	1.7	3	3.4	
Annual average gross income in Euros	Median	IQR	Median	IQR	Median	IQR	Median	IQR	Median	IQR	p value
Municipalities with available data	8,559	7,865–9,381	8,838	8,338–9,569	8,442	7,865–9,213	8,480	7,780–9,306	8,461	7,665–9,373	0.003^b^
Municipalities with no data	Number	%	Number	%	Number	%	Number	%	Number	%	p value
	9	1.1	3	2.1	3	1.2	3	1.0	0	0.0	NA
Gini coefficient	Median	IQR	Median	IQR	Median	IQR	Median	IQR	Median	IQR	p value
Municipalities with available data	0.31	0.29–0.34	0.32	0.29–0.34	0.31	0.29–0.33	0.31	0.29–0.34	0.32	0.30–0.34	0.204^b^
Municipalities with no data	Number	%	Number	%	Number	%	Number	%	Number	%	p value
	12	1.5	3	2.1	4	1.5	5	1.7	0	0.0	NA
Province	Number	%	Number	%	Number	%	Number	%	Number	%	p value
Granada	174	22.2	0	0.0	3	1.2	105	35.4	66	75.9	< 0.001^c^
Seville	106	13.5	20	14.2	54	20.8	31	10.4	1	1.1	
Almeria	103	13.1	51	36.2	35	13.5	17	5.7	0	0.0	
Malaga	103	13.1	0	0.0	70	26.9	32	10.8	1	1.1	
Jaen	97	12.4	0	0.0	16	6.2	67	22.6	14	16.1	
Huelva	80	10.2	56	39.7	24	9.2	0	0.0	0	0.0	
Cordova	77	9.8	10	7.1	41	15.8	23	7.7	3	3.4	
Cadiz	45	5.7	4	2.8	17	6.5	22	7.4	2	2.3	

IQR: interquartile range; MDR: multidrug resistant; NA: not applicable; sSIR: smoothed standardised incidence ratio.

^a^ p-value of Pearson’s Chi-squared test.

^b^ p-value of Kruskal–Wallis H test.

^c^ p-value of Fisher’s exact test.

Data are presented as absolute frequency (n) and relative frequency (%) for qualitative variables, and as median and IQR for quantitative variables. Georeferenced data were missing in 22.0% of cases. As a result, at the municipality level, only 4,982 cases were considered including 2,203 cases of healthcare-associated infections by extended-spectrum β-lactamase producing Enterobacterales, 1,377 by carbapenemase-producing Enterobacterales, 866 by meticillin-resistant *Staphylococcus aureus*, 486 by multidrug-resistant *Acinetobacter baumanii* and 232 by multidrug-resistant *Pseudomonas aeruginosa*.

**Figure 2:**
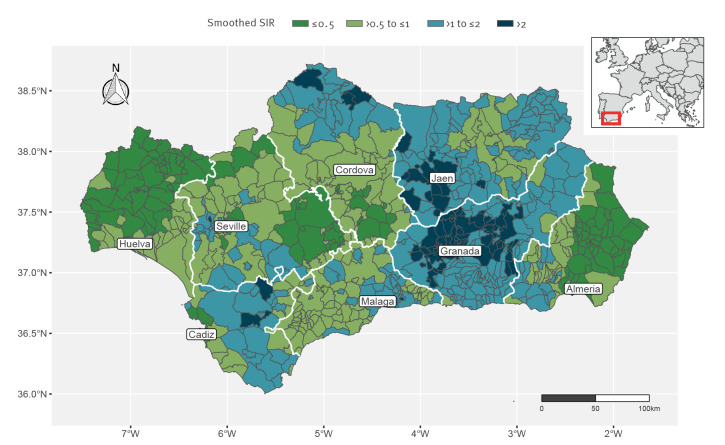
Spatial distribution of the smoothed standardised incidence ratio (SIR) of healthcare-associated infections by multidrug-resistant bacteria at the municipality level, Andalusia, Spain, 2014–2021 (n = 785 municipalities)

**Figure 3:**
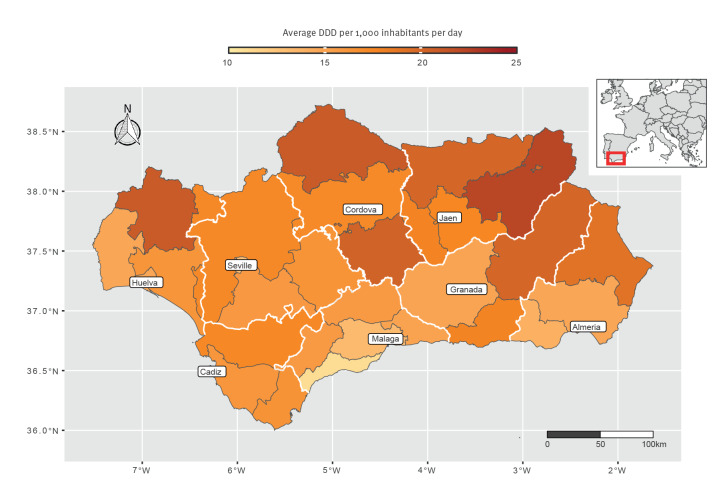
Spatial distribution of the outpatient antibiotic consumption in defined daily doses at the health district level, Andalusia, Spain, 2014–2021 (n = 33 health districts)

Global Moran’s I was 0.27, (p = 0.001), indicating a positive spatial autocorrelation. The SIR was calculated in 99.1% of municipalities (n = 778) and then smoothed to account for such correlation. Values of the sSIR ranged from 0.24 to 3.84, denoting great geographical variation: between areas with the highest incidence, mainly in the province of Granada, and those with the lowest incidence, mainly in the province of Huelva, there was a 15-fold difference (Figure 2 and Interactive content). When analysing the three most frequent mechanisms of resistance, this variation was markedly higher for CPE than for MRSA and ESBL-producing Enterobacterales as depicted in the Supplementary Material S4. However, the outpatient antibiotic consumption by health district exhibited a different spatial distribution, with the northern districts in Jaen (Jaen North-East = 22.3 DDD per 100,000 inhabitants per day), Huelva (Huelva North = 21.0) and Cordova (Cordova North = 21.0) presenting the highest values (Figure 3); this is also described in the Supplementary Material S5.

In the figure, white lines indicate provinces and grey lines indicate municipalities.

DDD: defined daily doses.

In the figure, white lines indicate provinces and grey lines indicate health districts.

In the bivariate analysis (Table 3), municipalities were categorised as having a very low (sSIR ≤ 0.5), low (sSIR > 0.5 to ≤ 1), high (sSIR > 1 to ≤ 2) and very high incidence (sSIR > 2); all cases were accounted for in these estimations, including the ones related to an outbreak. Municipalities with high and very high incidence showed significantly higher values in the deprivation index (p = 0.003), suggesting a direct albeit weak relationship between deprivation and the incidence of HAIs by MDR bacteria. Of note, this association did not change (p = 0.006) after excluding cases associated to an outbreak, as shown in Supplementary Material S6. Average gross income per capita was also significantly associated to the sSIR (p = 0.003), although income differences were only apparent between municipalities with very low incidence (where income values showed a greater dispersion) and the rest of municipalities. Interestingly, no differences were found according to the degree of urbanisation (p = 0.322), as represented in Supplementary Material S7. Consistent with the individual-level description, the province to which municipalities belonged was strongly associated with the sSIR (p < 0.001).

## Discussion

In this study, which analysed all HAIs by MDR bacteria notified to an epidemiological surveillance system during 8 years (2014–2021), we found HAIs were mostly caused by *Klebsiella* spp., *S. aureus* and *E. coli*, and affected more frequently men and patients over 60 years of age. HAIs due to ESBL-producing Enterobacterales and CPE showed a concerning increase. However, an exception to the temporal distribution occurred in 2020. That year coincided with the onset of the COVID-19 pandemic, which radically shifted the attention of surveillance towards a new pathogen capable of overburdening healthcare systems. We believe that this reason justifies the decrease in the reported cases of other notifiable HAIs and the subsequent compensatory increase during 2021 (also attributable to the impact of COVID-19 on antimicrobial resistance [28]). Nevertheless, the efforts made by SVEA professionals to retake surveillance of MDR organisms during the second year of the pandemic deserve a mention. That year aside, the constant upward trend of infections and colonisation by CPE outlines a scenario of limited therapeutic options and should suffice to alert epidemiological surveillance and to guide measures aimed at reducing their transmission.

Our findings are somewhat similar to those based on other epidemiological surveillance systems. In the United States (US), the National Healthcare Safety Network (NHSN) collects data on healthcare safety including HAIs from more than 5,000 facilities. Between 2011 and 2017, the main causative agents of HAIs were *E. coli*, *S. aureus* and *Klebsiella* spp., with *P. aeruginosa* becoming the fourth in frequency, and the proportion of carbapenem-resistant isolates increased substantially in *Enterobacter* spp. [29,30]. In the neighbouring country, the Canadian Nosocomial Infection Surveillance Programme (CNISP) reported that, although CPE colonisation rates significantly increased from 2014 to 2018 in acute care hospitals, infection rates remained stable (despite reaching their highest value in 2018) [31]. On the other hand, MRSA bloodstream infections increased [31]. A 20-year study describing antimicrobial resistance in Japan found an increase in the number of infections by carbapenem-resistant Enterobacterales since 2015, paired with a continuous decrease in that of MDR *A. baumannii* [32]. In Japan, prevalence of carbapenem resistance in *K. pneumoniae* isolates has been observed to be much lower than in Spain, while that of meticillin-resistance in *S. aureus* is among the highest in the world [32]; thus, caution is advised when comparing the epidemiology of MDR organisms across countries.

The role of long-term care facilities has gained interest in surveillance of HAIs. We did not find any notable characteristics in cases whose infection originated in long-term care facilities, other than *E. coli* being a more frequent causative agent than *S. aureus* (data not shown). This observation was similar to that of a laboratory-based 11-year study published in 2018, which analysed clinical isolates from nursing home patients in Switzerland, where *E. coli* was the most frequent agent identified [33]. The authors of that study reported that the proportions of extended-spectrum cephalosporin-resistant *E. coli* (that can be considered as proxy for to ESBL-producing) and carbapenem-resistant *P. aeruginosa* isolates notably increased over time in contrast to those of MRSA, which decreased. The main pathogen involved in urinary tract infections found between 2013 and 2017 in residents of US nursing homes was again *E. coli* [34] (probably reflecting its paramount importance as a uropathogen) and up to 36% of all infections were associated to antibiotic-resistant bacteria [34]. Together with the small number of cases of HAIs from long-term care facilities in our study (n = 131), this suggests that SVEA data underestimate the incidence of infections by MDR bacteria acquired at nursing homes.

Regarding antibiotic consumption, we found no previous work on HAIs by MDR bacteria suitable for comparison from a design standpoint. However, two ecological studies that examined the influence of antibiotic consumption on the incidence of *Clostridioides difficile* – a bacterium not necessarily MDR but nonetheless strongly related to antibiotic use – infection are worth mentioning. One used total outpatient antibiotic consumption in Wales [35] whereas the other used hospital consumption of high-risk antibiotics in Canada [36]. Both failed to find a clear association; thus, other factors may be involved. In terms of infection prevention and control – a much needed complement to antimicrobial stewardship – there is still room for improvement in Andalusia, especially regarding hand hygiene practices, with a compliance below 70% during the study period as shown in Supplementary Material S8.

We also provided SDoH potentially associated with the presence of MDR bacteria, identifying a possible direct relationship between the deprivation index and the sSIR. This suggests that lower educational level and worse employment conditions might entail a higher risk of developing HAIs by MDR bacteria at the municipality level. To our knowledge, only one ecological study had evaluated this outcome focusing on SDoH: a spatial analysis of healthcare- and community-associated MRSA strains in London (United Kingdom) adjusting by socioeconomic deprivation [37]. Contrary to our finding, Tosas Auguet et al. observed a substantial spatial heterogeneity in social and material deprivation across small areas of London. Nevertheless, they did find that healthcare-associated strains were associated with household deprivation. Unfortunately, the absence of accessible databases including social and environmental factors limits research based on epidemiological surveillance data [38,39]. In our opinion, such variables should be collected in detail in future studies on this topic.

In Andalusia, the unexpected high heterogeneity of incidence of HAIs by MDR bacteria can nevertheless not solely be explained by geographical differences. We believe that under-notification also contributed, with some areas displaying a much greater capacity of collecting and notifying cases than others (data not shown; the proportion of missing data in certain variables supports this hypothesis). Because data are reported by hand, such a capacity relies on the adequate coverage by epidemiologists across health districts; it might also be influenced by the workload and organisational aspects of each centre. Besides human resources, we propose two possible paths forward: in the short term, to compare diseases reported at smaller levels of analysis within comparable spatial units; in the medium to long-term, to actively homogenise the notification process by automating microbiological results and data from clinical records. Epidemiological surveillance systems could be an invaluable source of scientific information but standardised notification, as recommended in the European Centre for Disease Prevention and Control (ECDC) framework for data quality monitoring and surveillance system evaluation [40], is needed to fulfil their potential. How these pitfalls, certainly not unique to Spain [41], are dealt with may define upcoming research.

### Limitations

This study presents several limitations. First, information retrieved from the SVEA registries was incomplete (with missing data exceeding 50% of cases in some variables), partially due to heterogeneity in manual data collection by epidemiological surveillance professionals; also, it overrepresents hospitals as the infection source. Second, the selected causative agents and mechanisms of resistance were not homogeneous (e.g. MRSA and CPE), so their spatial and temporal distribution might differ. Third, despite studying the most frequent MDR bacteria, certain agents and mechanisms of resistance were not included (e.g. bacteria resistant to tetracyclines or fluoroquinolones); of note, we did not analyse vancomycin-resistant enterococci (VRE), which during the study period were considered infrequent in Spain [42,43] and which do not have a specific category in the SVEA protocol (HAIs by VRE may be notified as re-emerging or unusual microorganisms, but these could be underreported). Fourth, the distinction in terms of age between adult and paediatric intensive care units in the study region does not have an established cutoff point. However, more than 99% (838/844) of cases diagnosed at adult intensive care units were aged 18 years or above. Fifth, regarding the SDoH, data from a single year were obtained for each variable. While this decision helped avoid reverse causality bias, it failed to capture the dynamic relationship between social factors and antimicrobial resistance. Sixth, the use of the deprivation index in this study was not ideal. Its validity is higher in urban than in rural areas [17], which we tried to partially address by further including the degree of urbanisation; moreover, it was developed with census sections as the unit of analysis but we used it at the municipality level, incurring in an aggregation bias. Seventh, information on underlying diseases was not available, resulting in a less comprehensive description than desirable. We attempted to mitigate this deficit of clinical data by collecting socioeconomic variables. Eighth, although incidence rates were standardised by age and sex, we did not verify the proportionality of specific rates per subgroup between municipalities. Ninth, a discrepancy existed in the geographic unit of analysis. Outpatient antibiotic consumption was studied by health district because it was not feasible to assess such data at the municipality level.

## Conclusion

In this epidemiological surveillance-based study, a thorough description of HAIs by MDR bacteria in Andalusia, Spain, was presented. Data interpretation was limited by potential under-reporting and lack of data completion. We hope that our findings may help epidemiological surveillance systems overcome such pitfalls to control the exorbitant increase of HAIs by MDR bacteria in our environment. This study also underscores the need for public health measures to control antimicrobial resistance. We recommend reinforcing antimicrobial stewardship programmes, increasing compliance with standard and contact precautions, and implementing active microbiological surveillance in high-risk settings.

In addition, this study is among the first to pave the way for investigating the role of social factors in the acquisition and transmission of HAIs by MDR bacteria beyond *Mycobacterium tuberculosis* (in which transmission is known to be associated with socioeconomic status [44,45]). We found that deprivation index at the municipality level was associated with the incidence of these infections, leading us to suggest that SDoH could influence the spread of resistance in other pathogens.

## Supplementary Data

12191000 Supplementary Material
