# Supplementary material for: Healthcare-associated infections by multidrug-resistant bacteria in Andalusia, Spain, 2014 to 2021
Source: Euro Surveill. 2023 Sep 28;28(39):2200805. doi: 10.2807/1560-7917.ES.2023.28.39.2200805 (PMC10540512; doi:10.2807/1560-7917.ES.2023.28.39.2200805)
Supplement: Supplementary Material [file 2200805_SupplementaryMaterial.pdf]

This supplementary material is hosted by *Eurosurveillance* as supporting information alongside the article Healthcare-associated infections by multidrug-resistant bacteria in Andalusia, Spain, 2014 to 2021, on behalf of the authors, who remain responsible for the accuracy and appropriateness of the content. The same standards for ethics, copyright, attributions and permissions as for the article apply. Supplements are not edited by *Eurosurveillance* and the journal is not responsible for the maintenance of any links or email addresses provided therein.

Supplementary Material S1. Temporal distribution of cases of healthcare-associated infections by multidrug-resistant bacteria, according to the causative agent.

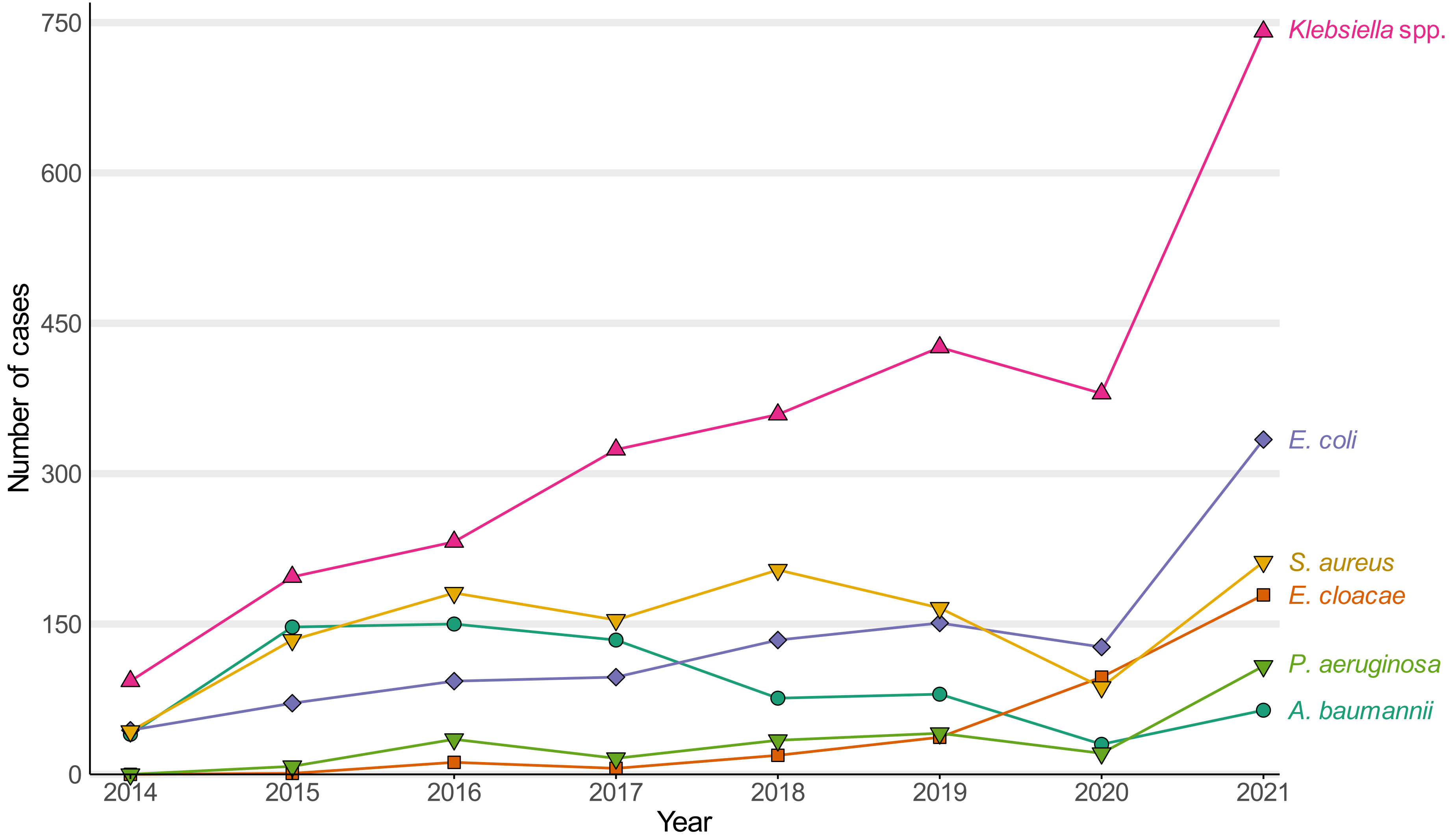

Supplementary Material S2. Basic demographic features of Andalusia, Spain, 2014 to 2021.

S2a. Temporal change in the population distribution, by province and age group.

Stratum	Population (2014)	Population (2021)	Temporal change
**Province**			
Almeria	701,688	731,792	+30,104 (+4.3%)
Cadiz	1,240,175	1,245,960	+5,785 (+0.5%)
Cordova	799,402	776,789	-22,613 (-2.9%)
Granada	919,455	921,338	+1,883 (+0.2%)
Huelva	519,229	525,835	+6,606 (+1.3%)
Jaen	659,033	627,190	-31,843 (-4.8%)
Malaga	1,621,968	1,695,651	+73,683 (+4.5%)
Seville	1,941,355	1,947,852	+6,497 (+0.3%)
**Age group, years**			
0-9	927,457	789,238	-138,219 (-14,9%)
10-19	883,031	956,010	+72,979 (+8.3%)
20-29	1,031,416	923,357	-108,059 (-10.5%)
30-39	1,358,796	1,109,748	-249,048 (-18,3%)
40-49	1,358,150	1,386,044	+27,894 (+2,1%)
50-59	1,091,605	1,282,786	+191,181 (+17.5%)
60-69	803,107	954,242	+151,135 (+18.8%)
70-79	571,241	649,045	+77,804 (+13.6%)
80-89	326,315	349,644	+23,329 (+7.1%)
≥ 90	51,187	72,293	+21,106 (+41.2%)
**Overall**	8,402,305	8,472,407	+70,102 (+0,8%)

Source: National Statistics Institute (INE).

S2b. Summary of the 10 most populated municipalities.

Municipality	DEGURBA classification	Population (2014)	Population (2021)	Share of the total population in Andalusia (2021)
Seville	City	696,676	684,234	8.1%
Malaga	City	566,913	577,405	6.8%
Cordova	City	328,041	322,071	3.8%
Granada	City	237,540	231,775	2.7%
Jerez de la Frontera	City	212,226	212,801	2.5%
Almeria	City	193,351	200,753	2.4%
Marbella	City	138,679	147,958	1.7%
Huelva	City	147,212	142,538	1.7%
Dos Hermanas	City	130,369	136,250	1.6%
Algeciras	City	117,974	122,982	1.5%
Total	-	2,768,981	2,778,767	32.8%

Source: National Statistics Institute (INE).

Supplementary Material S3. Temporal distribution of the outpatient antibiotic consumption in defined daily doses (DDD) at the health district level.

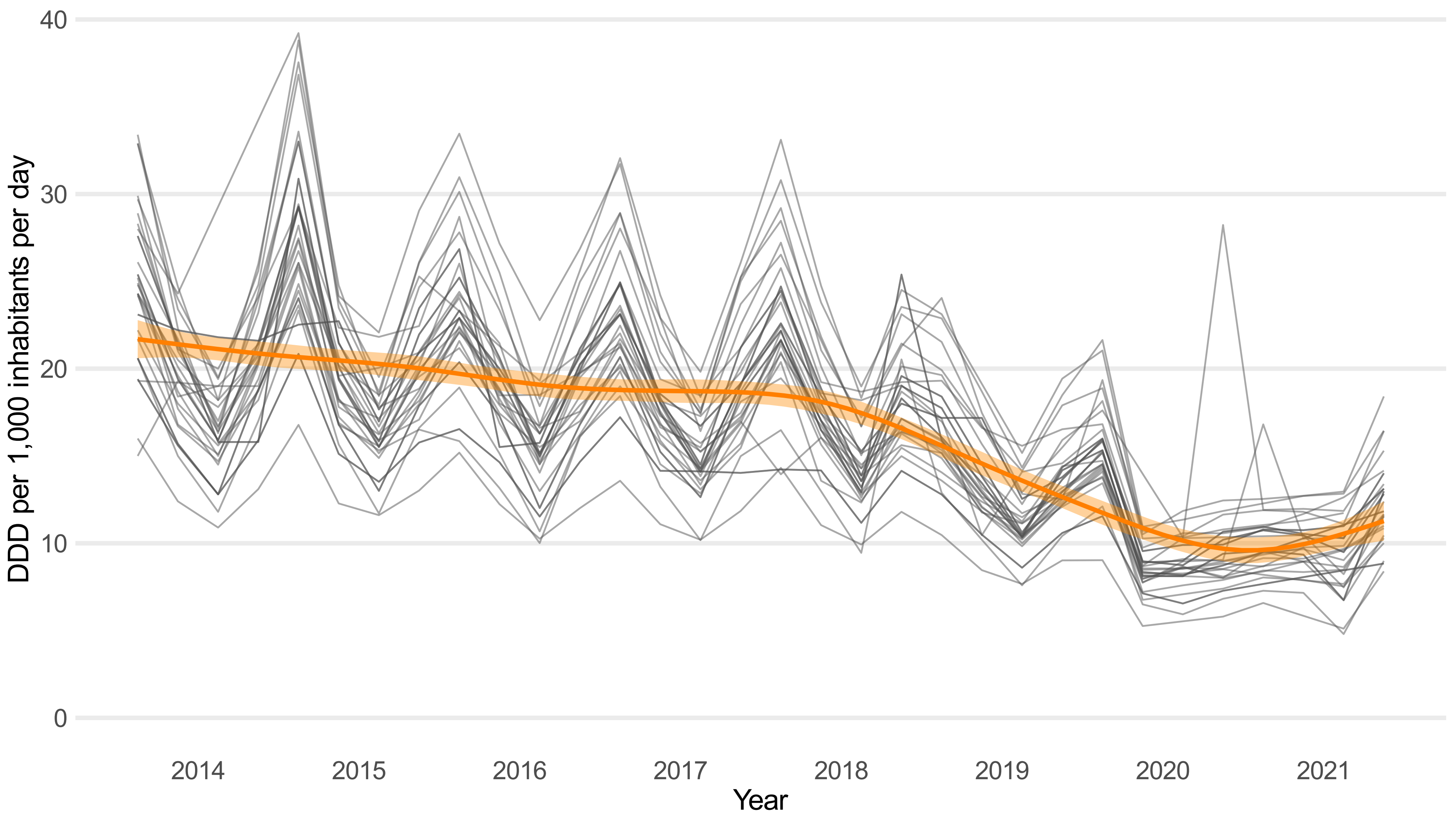

Supplementary Material S4. Spatial distribution of the smoothed standardised incidence ratio (SIR) of healthcare-associated infections by multidrug-resistant bacteria at the municipality level, according to the mechanism of resistance.

S4a. Extended-spectrum β-lactamase (ESBL) producing Enterobacterales.

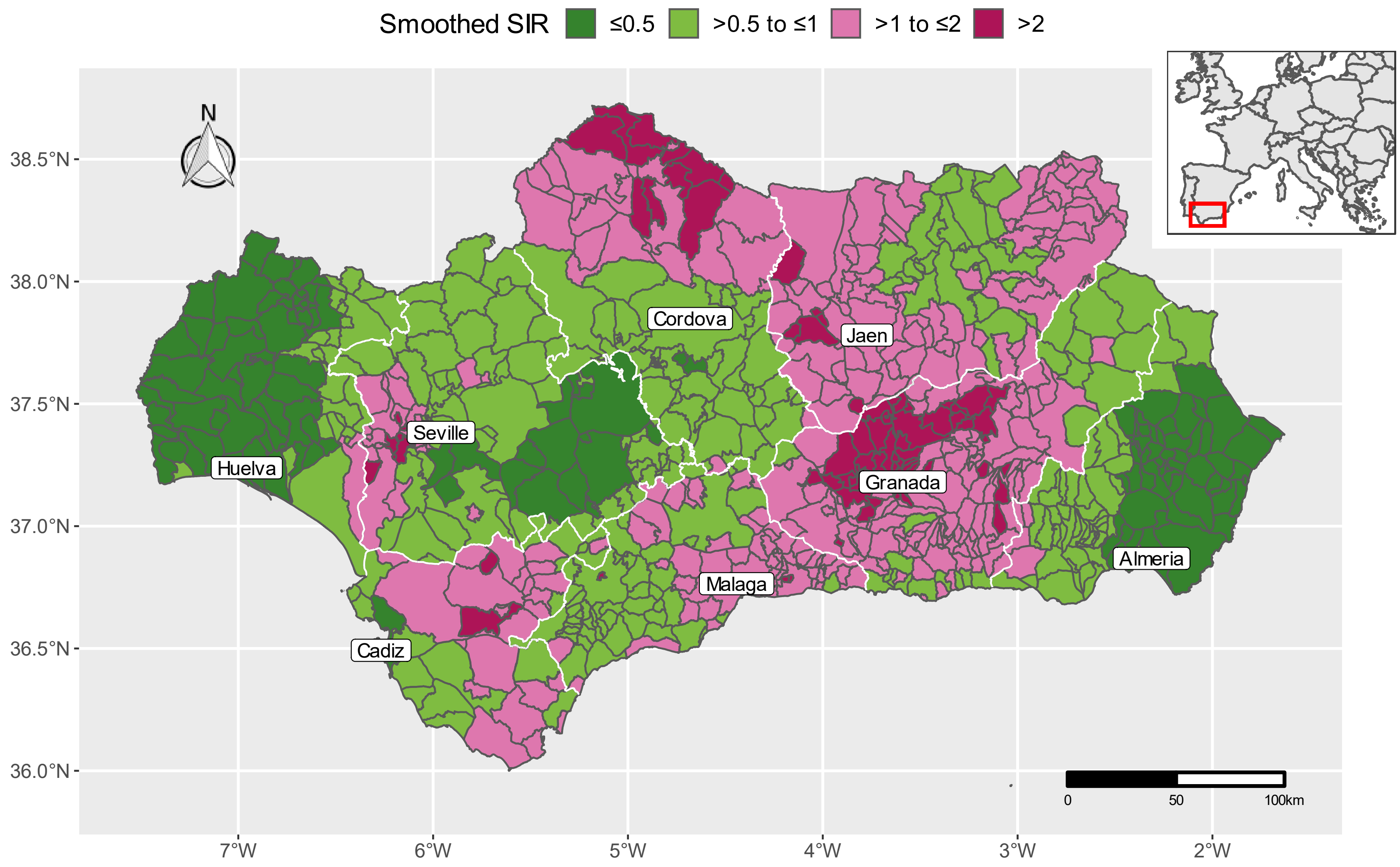

S4b. Carbapenemase-producing Enterobacterales (CPE).

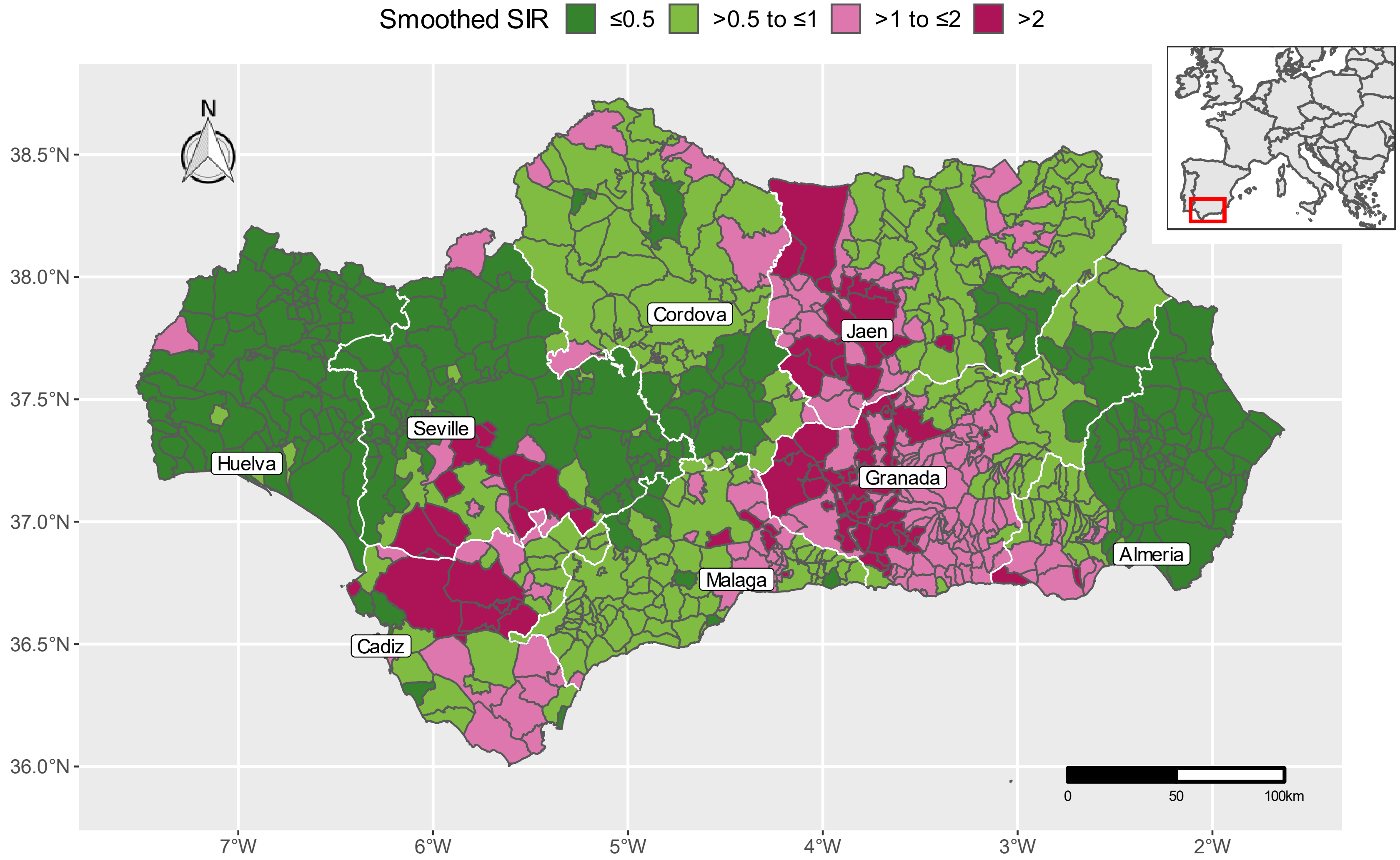

S4c. Methicillin-resistant *Staphylococcus aureus* (MRSA).

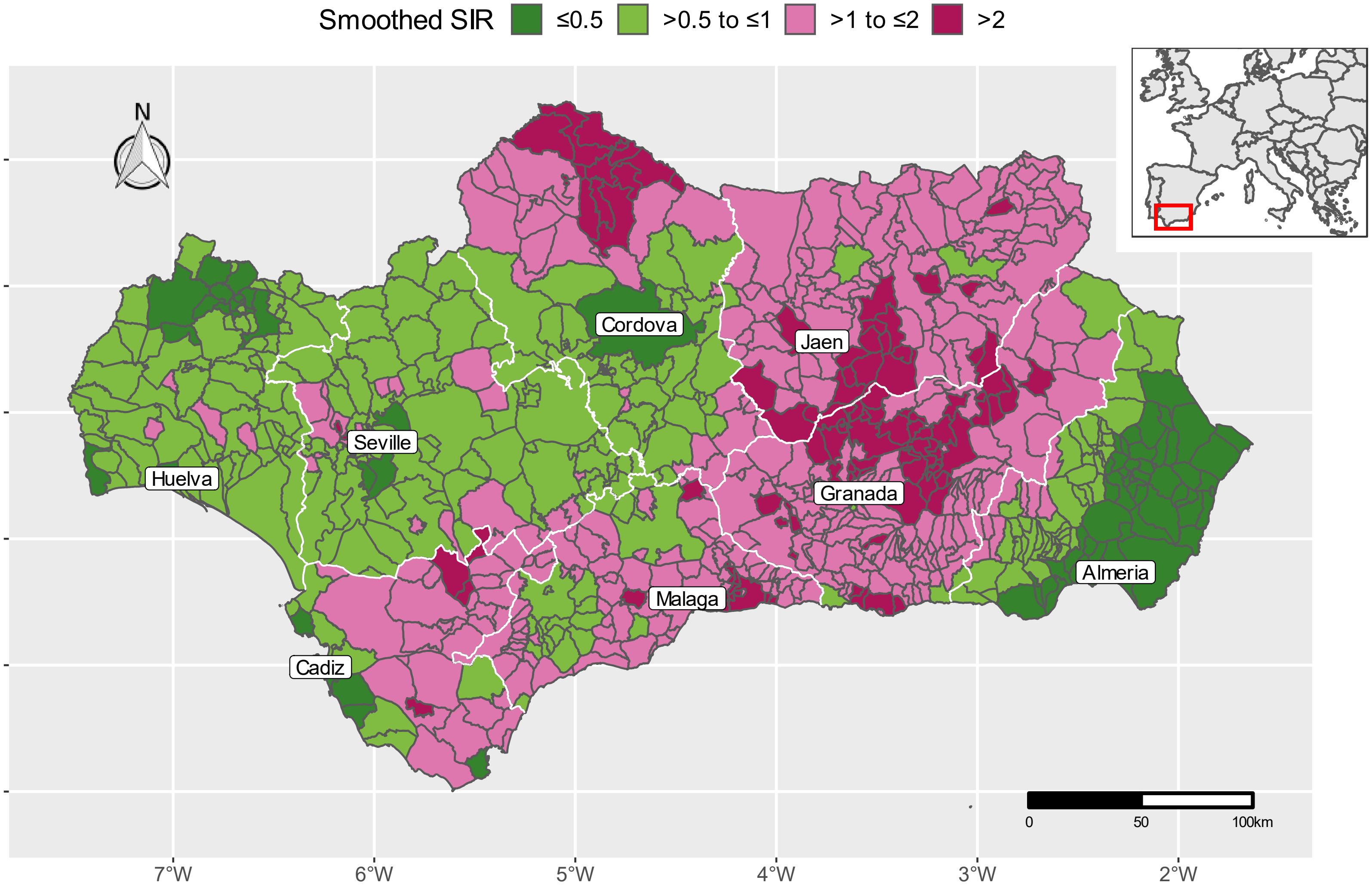

Supplementary Material S5. Outpatient antibiotic consumption in defined daily doses (DDD), stratified by year and health district.

Stratum	Outpatient antibiotic consumption, in DDD per 100,000 inhabitants per day, x (s)
**Year**	
2014	20.2 (4.5)
2015	21.1 (5.7)
2016	19.1 (4.5)
2017	18.6 (4.3)
2018	18.3 (4.5)
2019	13.8 (3.2)
2020	10.7 (3.7)
2021	10.6 (2.4)
**Health district**	
Jaen North-East	22.3 (7.2)
Huelva North	21.0 (6.6)
Cordova North	21.0 (7.0)
Cordova South	20.4 (7.1)
Jaen North	20.1 (5.9)
Granada North-East	20.1 (4.6)
Almeria North	19.1 (5.4)
Granada South	17.8 (4.9)
Jaen South	17.8 (6.4)
Jaen	17.6 (6.0)
Jerez-Costa North-West and Sierra de Cadiz*	17.4 (4.5)
Aljarafe and Seville North*	17.2 (5.4)
Cordova and Guadalquivir*	17.2 (5.7)
Campo de Gibraltar	16.4 (4.4)
Osuna (Seville East)	16.3 (5.6)
Condado-Campina	16.2 (5.1)
Bahia de Cadiz-La Janda	16.2 (5.0)
Seville South	15.9 (5.4)
Serrania	15.8 (4.8)
Malaga North (La Vega)	15.8 (4.9)
Malaga East (Axarquia)	15.1 (4.4)
Granada and Metropolitano de Granada*	14.9 (4.4)
Huelva-Costa	14.7 (4.5)
Almeria	14.7 (4.9)
Poniente de Almeria	14.0 (4.4)
Malaga and Valle del Guadalhorce*	13.2 (3.5)
Seville	12.8 (4.8)
Costa del Sol	10.8 (3.0)

Data are presented as mean (x) and standard deviation (s) for quantitative variables. DDD: Defined daily doses. Asterisks (*) indicate pairs of health districts for which data are grouped together in the PIRASOA reports: Jerez-Costa North-West and Sierra de Cadiz; Aljarafe and Seville North, Cordova and Guadalquivir; Granada and Metropolitano de Granada; Valle del Guadalhorce and Malaga.

Supplementary Material S6. Socio-demographic characteristics of municipalities of cases' residence, excluding cases associated to an outbreak, by level of incidence of healthcare-associated infections by MDR bacteria, Andalusia, Spain, 2014–2021 (n = 785 municipalities).

Characteristic	All municipalities (n = 785)		Very low incidence sSIR ≤ 0.5 (n = 124)		Low incidence sSIR > 0.5 to ≤ 1.0 (n = 257)		High incidence sSIR > 1.0 to ≤ 2.0 (n = 312)		Very high incidence sSIR > 2.0 (n = 92)		p value
**Number of inhabitants**	Number	%	Number	%	Number	%	Number	%	Number	%	
1–999	216	27.8	43	35.0	50	19.8	85	27.4	38	41.3	<0.001[a]
1,000–4,999	305	39.2	43	35.0	102	40.3	127	41.0	33	35.9	
5,000–19,999	175	22.5	26	21.1	58	22.9	73	23.6	18	19.6	
20,000–49,999	53	6.8	5	4.1	33	13.0	14	4.5	1	1.1	
≥ 50,000	29	3.7	6	4.9	10	4.0	11	3.6	2	2.2	
No data	7	0.8	1	0.7	3	1.2	3	1.0	0	0.0	
**Degree of urbanisation**	Number	%	Number	%	Number	%	Number	%	Number	%	0.162[a]
Rural areas	532	67.8	87	70.2	157	61.1	222	71.2	66	71.7	
Intermediate density areas	208	26.5	29	23.4	85	33.1	72	23.1	22	23.9	
Cities	45	5.7	8	6.4	15	5.8	18	5.8	4	4.4	
**Deprivation index**	Median	IQR	Median	IQR	Median	IQR	Median	IQR	Median	IQR	0.006[b]
Municipalities with available data	1.29	0.82–1.68	1.19	0.71–1.59	1.24	0.87–1.60	1.36	0.89–1.73	1.47	0.65–1.94	
Municipalities with no data	Number	%	Number	%	Number	%	Number	%	Number	%	NA
	14	1.8	1	0.8	6	2.3	4	1.3	3	3.3	
**Deprivation index quintiles**	Number	%	Number	%	Number	%	Number	%	Number	%	1.000[c]
Q1 (least deprived)	4	0.5	1	0.8	1	0.4	2	0.6	0	0.0	
Q2	20	2.5	2	1.6	8	3.1	8	2.6	2	2.2	
Q3	44	5.6	9	7.3	11	4.3	13	4.2	11	12.0	
Q4	126	16.1	29	23.4	37	14.4	47	15.1	13	14.1	
Q5 (most deprived)	577	73.5	82	66.1	194	75.5	238	76.3	63	68.5	
No data	14	1.8	1	0.8	5	1.9	5	1.6	3	3.3	
**Annual average gross income in Euros**	Median	IQR	Median	IQR	Median	IQR	Median	IQR	Median	IQR	0.027[b]
Municipalities with available data	8,559	7,865-9,382	8,772	8,192–9,523	8,528	7,932-9,395	8,473	7,787-9,291	8,478	7,665-9,551	
Municipalities with no data	Number	%	Number	%	Number	%	Number	%	Number	%	NA
	9	1.1	3	2.4	4	1.6	2	0.6	0	0.0	
**Gini coefficient**	Median	IQR	Median	IQR	Median	IQR	Median	IQR	Median	IQR	0.014[b]
Municipalities with available data	0.31	0.29–0.34	0.32	0.30–0.34	0.31	0.29–0.33	0.31	0.29–0.34	0.32	0.30–0.34	
Municipalities with no data	Number	%	Number	%	Number	%	Number	%	Number	%	NA
	12	1.5	3	2.4	5	1.9	4	1.3	0	0.0	
**Province**	Number	%	Number	%	Number	%	Number	%	Number	%	< 0.001[c]
Granada	174	22.2	0	0.0	3	1.2	96	30.8	75	81.5	
Seville	106	13.5	16	12.9	70	27.2	18	5.8	2	2.2	
Almeria	103	13.1	51	41.1	32	12.5	20	6.4	0	0.0	

Malaga	103	13.1	0	0.0	55	21.4	46	14.7	2	2.2	
Jaen	97	12.4	0	0.0	10	3.9	76	24.4	11	12.0	
Huelva	80	10.2	52	41.9	26	10.1	2	0.6	0	0.0	
Cordova	77	9.8	1	0.8	48	18.7	27	8.7	1	1.1	
Cadiz	45	5.7	4	3.2	13	5.1	27	8.7	1	1.1	

Data are presented as absolute frequency (n) and relative frequency (%) for qualitative variables, and as median and interquartile range (IQR) for quantitative variables. MDR: multidrug resistant; NA: not applicable; sSIR: smoothed standardised incidence ratio. [a] p-value of Pearson's Chi-squared test. [b] p-value of Kruskal–Wallis H test. [c] p-value of Fisher's exact test.

Supplementary Material S7. Smoothed standardised incidence ratio (SIR) of healthcare-associated infections by multidrug-resistant bacteria, according to the degree of urbanisation.

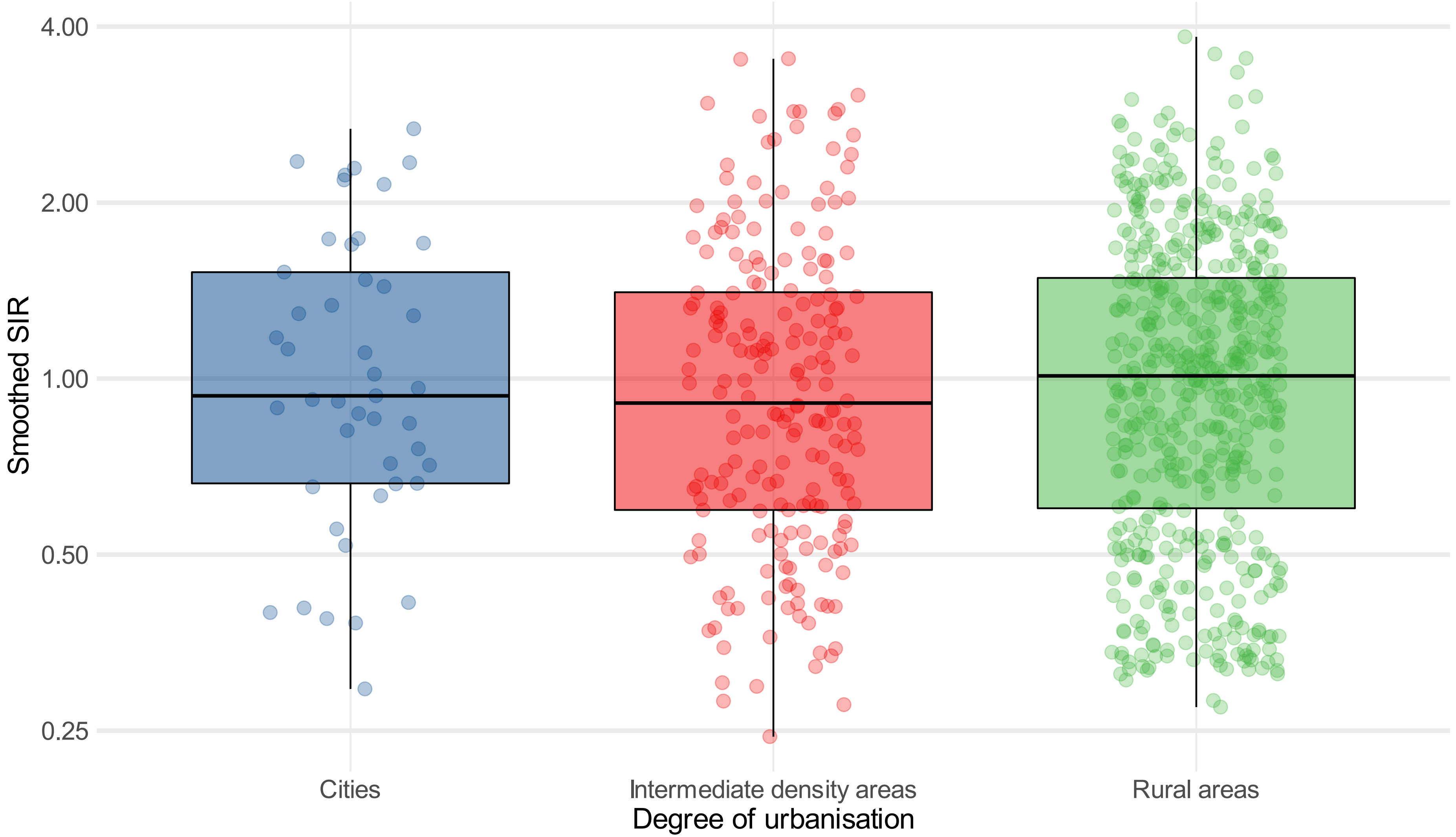

Supplementary Material S8. Overview of compliance with infection prevention and control practices in the hospitals of the public healthcare system of Andalusia, Spain, stratified by year.

Parameter	2014	2015	2016	2017	2018	2019	2020	2021
**Hand hygiene**	56.3 (19.8)	56.2 (19.9)	54.6 (21.9)	61.6 (17.4)	60.2 (17.3)	64.0 (19.0)	72.6 (22.7)	68.0 (24.1)
**Contact precautions**	68.7 (31.3)	73.7 (28.7)	78.9 (26.8)	82.0 (23.5)	84.4 (19.8)	84.5 (19.4)	84.0 (23.5)	88.5 (16.9)
**Neumonía Zero**	76.7 (30.4)	83.3 (27.3)	88.7 (18.6)	87.0 (20.9)	87.1 (20.1)	87.5 (18.0)	89.5 (15.9)	86.7 (15.9)
**Bacteriemia Zero**	78.8 (29.6)	86.1 (23.1)	91.7 (17.4)	92.6 (14.6)	90.1 (21.0)	89.6 (22.6)	95.0 (10.8)	92.9 (19.3)

Data are presented as mean (x) and standard deviation (s) for quantitative variables, which are all measured in a scale ranging from 0 (no compliance) to 100 (perfect compliance).
Data source: quarterly reports of the PIRASOA programme, 2014 to 2021 (please see reference no. 18 in the manuscript).

In the table above, hand hygiene refers to observations assessing hand hygiene practices, performed by trained observers. Contact precautions refers to compliance with recommendations of contact precautions to reduce the transmission of multidrug-resistant bacteria (e.g., carbapenemase-producing Enterobacterales). “Neumonía Zero” is a nationwide project developed by the Spanish Society of Intensive Medicine (SEMICYUC) in 2011 in order to reduce the incidence of ventilator-associated pneumonia (https://semicyuc.org/proyecto-neumonia-zero/). Designed from a patient safety standpoint, it includes a series of indicators that are assessed periodically. The aggregate indicator shown summarises the overall compliance. Finally, “Bacteriemia Zero” is also a nationwide project developed by the same society (SEMICYUC) in 2009 in order to reduce the incidence of catheter-associated bloodstream infection (https://semicyuc.org/bacteriemiazero/). Again, the aggregate indicator shown summarises the overall compliance.
